# Risk factors for drug-related problems in a general hospital: A large prospective cohort

**DOI:** 10.1371/journal.pone.0230215

**Published:** 2020-05-05

**Authors:** Valdjane Saldanha, Ivonete Batista de Araújo, Sara Iasmin Vieira Cunha Lima, Rand Randall Martins, Antonio Gouveia Oliveira

**Affiliations:** 1 Graduate Program in Pharmaceutical Sciences, Centro de Ciências da Saúde, Federal University of Rio Grande do Norte, Natal, RN, Brazil; 2 Pharmacy Department, Centro de Ciências da Saúde, Federal University of Rio Grande do Norte, Natal, RN, Brazil; Mahidol University, THAILAND

## Abstract

**Objective:**

To identify risk factors for potential Drug-Related Problems (DRP) at admission in hospitalized patients.

**Methodology:**

Prospective cohort study conducted in adults patients hospitalized (May 2016 to May 2018) in a general tertiary care hospital in Brazil. Potential DRP were detected by daily review of 100% of electronic medication orders by hospital pharmacists and classified by the Pharmaceutical Care Network Europe classification system (PCNE version 6.2). For the identification of risk factors of potential DRP, backward stepwise logistic regression was used to identify the set of independent predictors among over 120 variables collected in the initial 48 hours after admission in a training set consisting of 2/3 of the study population. The model was validated in the remaining sample.

**Results:**

The study population consisted of 1686 patients aged 52.0+/- 18.3 years-old, 51.4% females, with a median length of stay of 3.24 days, and 4.5% in-hospital mortality. The cumulative incidence of potential DRP was 14.5%. Admission for elective surgery and main diagnosis of disease of the circulatory system were associated with reduced risk of DRP (OR 0.41 and 0.57, respectively, p<0.05). The independent risk factors of DRP are heart rate ≥ 80 bpm (OR 1.41, p = 0.05), prescription of more than seven drugs in day 2 (OR 1.63, p = 0.05), prescription in day 1 of drugs of the Anatomical Therapeutic Chemical Code (ATC) class A (alimentary tract and metabolism, OR 2.24, p = 0.003), prescription in day 2 of two or more ATC class A drugs (OR = 3.52, p<0.001), and in day 1 of ATC class J drugs (antiinfectives for systemic use, OR 1.97, p = 0.001). In the validation set, the c-statistic of the predictive model was 0.65, the sensitivity was 56.1% and the specificity was 65.2%.

**Conclusion:**

This study identified seven independent risk factors of potential DRP in patients hospitalized in a general hospital that have fair predictive performance for utilization in clinical practice.

## Introduction

Drug-related problems (DRP) are defined as “events or circumstances involving drug therapy that actually or potentially interfere with desired health outcomes” [1]. The definition of DRP is comprehensive, encompassing the concepts of adverse events, which may be preventable or not, adverse reactions, which are not preventable, and medication errors, which include prescription errors, drug dispensing errors, and drug administration errors [2]. DRP that are detected are classified as potential, if they have the prospective of causing harm to the patient regardless of whether a harm was actually observed, and manifest if they did have caused harm to the patient [1]. Therefore, potential DRP do not necessarily imply patient injury: medication errors, for example, uncommonly lead to patient harm.

Events related to medication use are common in adults [3] and children [4], at any level of care [5–8]. Problems involving medication are associated with a higher number of hospitalizations, long-term hospitalizations, admission to emergency services, additional visits to the doctor's office, additional prescriptions and death [9,10]. Therefore, the economic burden and morbimortality are evident [8,11,12]. As reported in previous studies, the cumulative incidence of DRP in hospitalized patients showed large variation, from 27.8% [13] to 81% [14], mainly in relation to the characteristics of the target population [14], the methodology adopted for their detection and their classification [15]. Several classification systems of DRP have emerged, among them the Pharmaceutical Care Network (PCNE), which has been the most widely used internationally in hospital clinical practice [16].

Several strategies for detecting DRP have been used to assist in the daily monitoring of drug therapy. According to the literature, these have included medical chart review, associated or not with electronic alerting systems [17], and the integration of pharmacists into the health care teams during ward rounds [18]. In pharmacoepidemiology, medical chart review is considered the gold standard [19] for improvement of the safety of the medication process [17, 20–22] and, from this process, to enable the identification of clinical and demographic characteristics that may be considered predictors of increased risk developing a DRP in hospitalized patients. The knowledge of risk factors of DRP for a population exposed to a multifactorial environment, such as the hospital, is necessary to assist in directing actions for the implementation of damage prevention strategies, offering quality and safety in health care and resource optimization.

Currently, little is known about risk factors for the development of DRP in the average patient in a general hospital. Risk factors for DRP have been specifically studied in elderly populations [23], patients from medical specialties such as cardiology [24,25] and paediatrics [26]. To the best of our knowledge, there has been a single report in the literature investigating risk factors and developing a risk score for DRP in adult patients at hospital admission in general wards, the study by Urbina et al [13], with a subsequent validation study by Ferrández et al. [27]. A 2015 study suggested a set of risk factors, but they were selected on the basis of expert opinions [28]. Therefore, the present study represents a further attempt to fill a knowledge gap regarding risk factors of potential DRP at admission in patients hospitalized in a general tertiary care hospital.

## Patients and methods

This was prospective observational cohort study conducted at Onofre Lopes University Hospital, a public hospital in Natal, Rio Grande do Norte, Brazil, which was approved by the institution's Research Ethics Committee with approval number 1.439.845. All patients gave written informed consent and no incentives were offered to participants.

To avoid bias due to seasonality, patient enrolment proceeded without interruption for 2 years from May 2016 to May 2018. Inclusion criteria were: patients aged 18 years or older, of both sexes, who were hospitalized for more than 24 hours in the departments of nephrology, urology, cardiology, surgery (general, neurological, cardiovascular and oncological), endocrinology, rheumatology, neurology, gastroenterology and psychiatry, and who were administered at least one medication. Patients admitted for diagnostic purposes only, who were on cancer chemotherapy regimens, transplanted patients, pregnant women and patients hospitalized in the intensive care unit were excluded.

In order to obtain the target sample size distributed evenly over the defined two-year patient accrual period, a random sample of patients was obtained by enrolling into the study all eligible patients admitted on two different days (consecutive or not) of each study week A randomization list of the two days in each study week when patient enrolment would occur was prepared before the study start using a random number generator.

All patients included in the study cohort were followed throughout their hospital stay from admission to discharge. Potential DRP were detected by daily review of 100% of the electronic medication orders distributed evenly by groups of 3 pharmacists/day from a total of 21 hospital pharmacists assigned to the project. Although the hospital has an electronic patient record and a computerized physician order entry system (without an integrated DRP warning system), only the latter was inspected for the detection of DRP. Each potential DRP identified by the pharmacists was then classified by a clinical pharmacist with 9 years’ experience using the Pharmaceutical Care Network Europe classification system (PCNE version 6.2), according to the type of problem and causes. In cases of doubt in the accurate classification, another pharmacist with more than 10 years’ experience was consulted and a consensual decision was reached. No evaluation of adverse reactions and dispensing errors was made because the potential DRP detection method based exclusively on medication order review did not provide data to verify such problems. All identified potential DRP were subsequently reported to the healthcare team through standard forms in use in the hospital for that purpose.

Patient demographic and clinical data were collected from all patients at admission, as well prescription data in the first and second hospitalization day. These data were collected from all patients who were admitted to the medical and surgical clinics. The patient data were collected by pharmacy students and the identification of potential DRP was done by pharmacists from the hospital pharmacy service. Patient-related variables were age, sex, race, hospitalization in the previous 12 months, body mass index, vital signs, type of admission (medical, elective surgery or emergency surgery), co-morbidities, International Disease Classification version 10 (WHO-CID 10) chapter of the main diagnosis, Charlson's comorbidity index, and routine laboratory data (haemoglobin, leukocytes, creatinine, albumin, alanine aminotransferase, and gamma-glutamyl-transpeptidase). Drug related variables were number of drugs prescribed, number of drugs prescribed in each Anatomical Therapeutic Chemical Code (ATC) therapeutic group, number of drugs by route of administration, presence of drug-drug interaction, drug-food interaction and drug incompatibility.

### Statistical analysis

The target sample size was defined at 1800 patients, which would afford 80% power at the two-tailed significance level of 5% to identify risk factors of DRP with an odds-ratio greater than 2.38 in variables with a prevalence of 5%, and with an odds-ratio greater than 1.64 in variables with a prevalence of 20%.

Stata 15 (Stata Corporation, College Station, TX, USA) was used in all analyses. All continuous predictor variables were dichotomized by constructing receiver operating characteristics (ROC) curves for each one and then selecting as cut-off the value corresponding to an approximately equal sensitivity and specificity for the occurrence of one or more DRP. For the identification of risk factors of DRP we used univariate logistic regression to test the association between a dependent binary variable, coded 0 if no DRP had been observed in a patient and 1 otherwise, with each demographic and clinical variable collected at admission and drug data collected on the first and second days of hospitalization. Only variables that occurred in more than 5% of the patients were analysed. Variables in which the test of the regression coefficient yielded a p-value greater than 0.20 were considered not associated. In an attempt to derive a predictive model that could be used as a risk stratification tool for DRP in patients hospitalized in a general tertiary-care hospital, the study population was randomly divided in a 2:1 ratio into a training set and a validation set. Using the training set, the variables identified in the previous univariate analysis were entered into a multivariate logistic regression model using a stepwise backward variable selection method retaining only those predictor variables associated with the outcome at the 5% significance level. Interactions were not tested and estimates relate to main effects only. Model fit in the training set was evaluated for discrimination by the area under the ROC curve (c-statistic) and for calibration with the Hosmer-Lemeshow test. The performance of the prediction model was assessed with the Brier score and with the analysis of the calibration plot and the calibration belt plot, obtained with the pmcalplot [29] and calibrationbelt [30] commands of Stata. Model sensitivity, specificity and predictive values positive and negative were computed. These statistics, as well as the c-statistic and calibration plots were then obtained from the validation set.

## Results

During the two years’ enrolment period, there were 14,527 hospitalization episodes. From these, 3,877 (25%) were the total number of admissions in the two week days selected at random. After assessment of exclusion criteria 2,026 patients were excluded and a further 23 patients declined participation in the study. In the remaining 1,803 hospitalization episodes, there were 1,686 distinct patients. Only the first hospitalization episode was considered in patients with multiple hospitalizations.

The mean age of the 1,686 unique patients representing the study population was 52.0 ± 18.3 years and 867 (51.4%) were female. Table 1 summarizes the main characteristics of the study population.

**Table 1:** Characteristics of the study population (n = 1686).

Variable	Descriptive statistics
Age, m(sd)	52.0 (18.3)
Female sex, n(%)	867 (51.4%)
Type of admission, n(%)	
medical	516 (30.6%)
elective surgery	1064 (63.1%)
urgent surgery	106 (6.29%)
Hospital department, n(%)	
Gastrenterology	283 (16.8%)
Cardiology	271 (16.1%)
Urology	270 (16.0%)
General surgery	196 (11.6%)
Nephrology	90 (5.34%)
Charlson comorbidity index, md (Q1-Q3)	3 (3–4)
Drug related problem, n(%)	245 (14.5%)
Length of stay in days, md(Q1-Q3)	3.24 (1.82–11.3)
In-hospital death, n(%)	72 (4.45%)

m: mean; md: median; Q1-Q3: first and third quartiles; sd: standard deviation.

The total number of potential DRP detected by the hospital pharmacists was 519 in 245 patients, corresponding to a cumulative incidence of potential DRP in the study cohort of 14.5% (95% confidence interval (CI) 12.9%– 16.3%). The average number of DRP among hospitalized patients was 0.31 ± 0.90 (95%CI 0.26–0.35) and among patients with potential DRP was 1.90 ± 1.46 (95%CI 1.72–2.09). A summary of the detected potential DRP in the four problem classes and according to causes is presented in Table 2.

**Table 2:** Classification of potential DRP according to the PCNE 6.2 classification system.

*PCNE*		*Distribution among potential DRPs*		*Distribution among patients*	
		n	%	n	%
Problems					
P1.1	No effect of drug treatment/ therapy failure	42	8.09	38	2.25
P1.2	Effect of drug treatment not optimal	248	47.8	175	10.38
P1.3	Wrong effect of drug treatment	5	0.96	4	0.24
P3.1	Drug treatment more costly than necessary	159	30.6	101	5.99
P4.2	Unclear problem/complaint. Further clarification necessary (please use as escape only)	65	12.5	53	3.14
Causes					
C1.1	Inappropriate drug (incl. contra-indicated)	5	0.96	4	0.24
C1.2	No indication for drug	5	0.96	5	0.30
C1.3	Inappropriate combination of drugs, or drugs and food	7	1.35	5	0.30
C1.4	Inappropriate duplication of therapeutic group or active ingredient	3	0.58	3	0.18
C1.7	More cost-effective drug available	2	0.39	2	0.12
C2.1	Inappropriate drug form	1	0.19	1	0.06
C3.2	Drug dose too high	65	12.52	60	3.56
C3.3	Dosage regimen not frequent enough	30	5.78	30	1.78
C4.2	Duration of treatment too long	157	30.25	99	5.87
C5.1	Inappropriate timing of administration and/or dosing intervals	80	15.41	69	4.09
C6.1	Prescribed drug not available	37	7.13	33	1.96
C6.2	Prescribing error (necessary information missing)	122	23.51	93	5.52
C8.1	Other cause	5	0.96	5	0.30

For the univariate analysis by logistic regression of the variables associated with the occurrence of one or more potential DRP, the following variables were excluded from the analysis because they were observed in less than 5% of the patients: body temperature > 37.5 C, administration of one or more medications of ATC classes D (dermatologicals), G (genito-urinary system and sex hormones), L (antineoplastic and immunomodulating agents), S (sensory organs) and V (various), all administration routes except oral, intravenous and subcutaneous, all ICD-10 chapters of main diagnosis except chapters II (neoplasms), IX (circulatory system), X (respiratory system), XI (digestive system) and XIV (genitourinary system).

**Fig 1:**
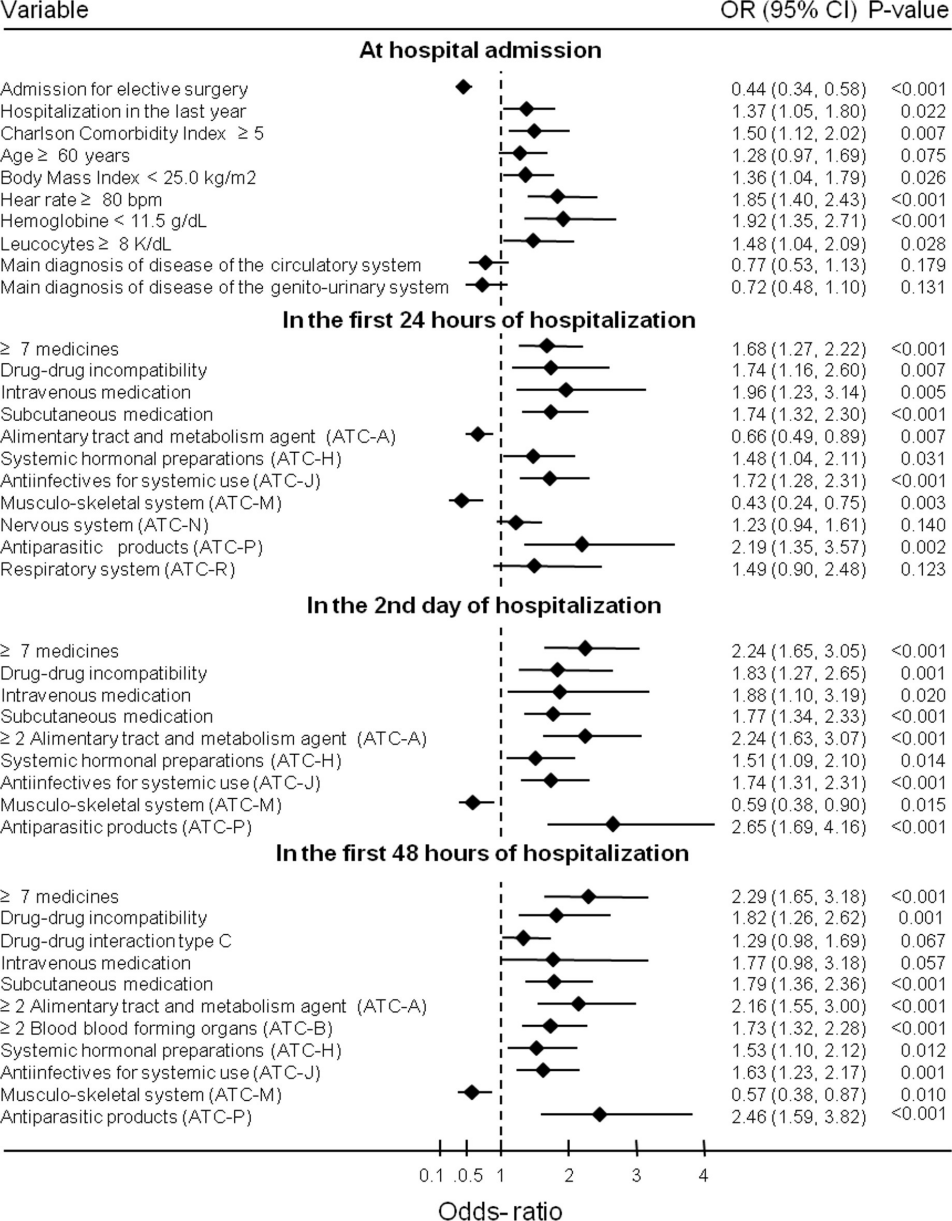
Univariate analysis of risk factors of drug related problems in patients hospitalized in a general hospital.

In univariate logistic regression analysis (Fig 1), the following patient-related variables collected at hospital admission were statistically significantly associated, at the 5% significance level, with an increased risk of occurrence of one or more potential DRP: hospitalization during the previous 12 months (OR 1.37), Charlson’s Comorbidity Index ≥5 (OR 1.50), Body Mass Index < 25.0 kg/m^2^ (OR 1.36), heart rate ≥ 80 beat per minute (OR 1.85), haemoglobin < 11.5g/dL (OR 1.92) and leukocytes ≥ 8 K/dL (OR1.48); hospitalization for elective surgery decreased the risk of potential DRP occurrence (OR 0.44).

In the analysis of the variables related to the drugs prescribed in the first 24 hours of hospitalization, the risk for potential DRP decreases when an ATC classification code A drug (alimentary tract and metabolism) was present in the prescription (OR 0.66) or an ATC code M drug (musculo-skeletal system, OR 0.43). Drug-related factors that increase the risk of DRP were prescription of ≥ 7 medications (OR 1.68), drug-drug incompatibility (OR 1.74), prescription of intravenous drug (OR 1.96) and subcutaneously (OR 1.74), prescription of ATC code H medications (systemic hormonal preparations, OR 1.48), administration of ATC code J drugs (antiinfectives for systemic use, OR 1.72), and administration of ATC code P drugs (antiparasitic medications, OR 2.19).

In the second day of hospitalization, risk factors are the same as those identified in the first 24 hours of hospitalization, but with different odds-ratios. The exception is the prescription of two or more alimentary tract and metabolism agents that increases the risk of potential DRP (OR 2.24).

The prescription of 2 or more alimentary tract and metabolism agent and of 2 or more agents that act in the blood and blood forming organs (ATC code B) in the first 2 days of hospitalization are factors of increased risk for the occurrence of potential DRP, in addition to the factors observed on the second day of hospitalization.

Table 3 shows the three most prescribed medications corresponding to each of the variables associated with the occurrence of one or more DRP identified in the univariate analysis.

**Table 3:** Most prescribed medications within each variable associated with the occurrence of one or more DRP in patients hospitalized in a general tertiary-care hospital.

Variable	Day 1		Day 2		First 48 hrs	
	n	%	n	%	n	%
≥ 7medications						
Dipyrone	784	89.4	889	88.7	1077	97.6
Metoclopramide	404	46.1	467	46.6	545	49.4
Ondansetron	363	41.4	434	43.3	537	48.7
Drug-drug interaction						
Pantoprazole	104	64.6	157	81.3	165	82.9
Onsansetron	26	16.1	33	17.1	36	18.1
Phenytoin	14	8.7	15	7.8	16	8.0
Intravenous medication						
Dipyrone	1030	71.5	1049	69.8	1105	71.6
Metoclopramide	611	42.4	609	40.5	643	41.7
Ondansetron	605	42.0	689	45.8	722	46.8
Subcutaneous medication						
Insulin	427	79.2	455	78.0	479	78.5
Enoxaparine	198	36.7	234	40.1	248	40.7
Heparin	46	8.5	55	9.4	60	9.8
Alimentary tract and metabolism agent (ATC-A)						
Metoclopramide	318	54.5				
Ondansetron	166	28.5				
Ranitidine	34	5.8				
≥ 2 Alimentary tract metabolism agent (ATC-A)						
Ondansetron			540	51.2	599	53.3
Metoclopramide			504	47.8	602	53.6
Omeprazole			399	37.8	435	38.7
≥ 2 Blood blood forming organs (ATC-B)						
Saline solution					299	44.0
Ringer lactate					227	33.4
Acetylsalicylic acid					202	29.7
Systemic hormonal preparations (ATC-H)						
Dexamethasone	84	34.9	96	32.9	96	33.1
Prednisone	58	24.1	63	21.6	60	20.7
Levothyroxine	50	20.7	51	17.5	51	17.6
Hydrocortisone	48	19.9	62	21.2	63	21.7
Antiinfectives for systemic use (ATC-J)						
Ceftriaxone	129	33.4	136	29.8	136	29.1
Cefazolin	90	23.3	136	29.8	146	31.2
Ciprofloxacin	69	17.9	70	15.3	71	15.2
Musculo-skeletal system (ATC-M)						
Tenoxicam	173	89.6	252	94.0	264	93.3
Alendronate	2	1.0	1	0.4	2	0.7
Cochicine	2	1.0	1	0.4	1	0.4
Nervous system (ATC-N)						
Dipyrone	728	93.3				
Paracetamol	29	3.7				
Clonidine	10	1.3				
Antiparasitic products (ATC-P)						
Metronidazol	64	69.6	72	70.6	75	67.6
Hydroxychloroquine	17	18.5	19	18.6	20	18.0
Ivermectine	7	7.6	3	2.9	9	8.1
Respiratory system (ATC-R)						
Dimenidrate	46	45.5				
Dexchlorpheniramine	6	5.9				
Fenoterol	6	5.9				

For the development of a clinical instrument predictive of the occurrence of one or more potential DRP in patients hospitalized in a general tertiary care hospital, the training set consisting of 1,124 patients was used to develop a multivariate model by logistic regression with stepwise variable selection of all the variables identified by univariate analysis. Haemoglobin and leucocyte count were excluded from the analysis because those data were missing in a large proportion of patients. Table 4 presents the variables that were independently associated with the occurrence of one or more potential DRP in multivariate logistic regression. The Aikake Information criterium was reduced from 899.6 in the full model with 37 variables to 865.8 in the final model with 7 variables. The pseudo R-squared was 0.09.

**Table 4:** Independent risk factors of DRP in patients hospitalized in a general tertiary care hospital.

Risk factor	Regression coefficient	Adjusted odds- ratio	95% confidence interval		p
Admission for elective surgery	-0.8924	0.41	0.29	0.59	<0.001
Main diagnosis of disease of the circulatory system	-0.5593	0.57	0.34	0.96	0.035
Hear rate ≥ 80 beats per minute	0.3433	1.41	1.00	2.00	0.053
≥ 7 medicines in day 2	0.4873	1.63	1.07	2.47	0.022
Alimentary tract and metabolism agent (ATC-A) in day 1	0.8058	2.24	1.31	3.84	0.003
≥ 2 Alimentary tract and metabolism agent (ATC-A) in day 2	1.2592	3.52	1.99	6.25	<0.001
Antiinfectives for systemic use (ATC-J) in day 1	0.6765	1.97	1.34	2.89	0.001
Regression constant	-2.9899	0.05	0.02	0.10	<0.001

**Fig 2:**
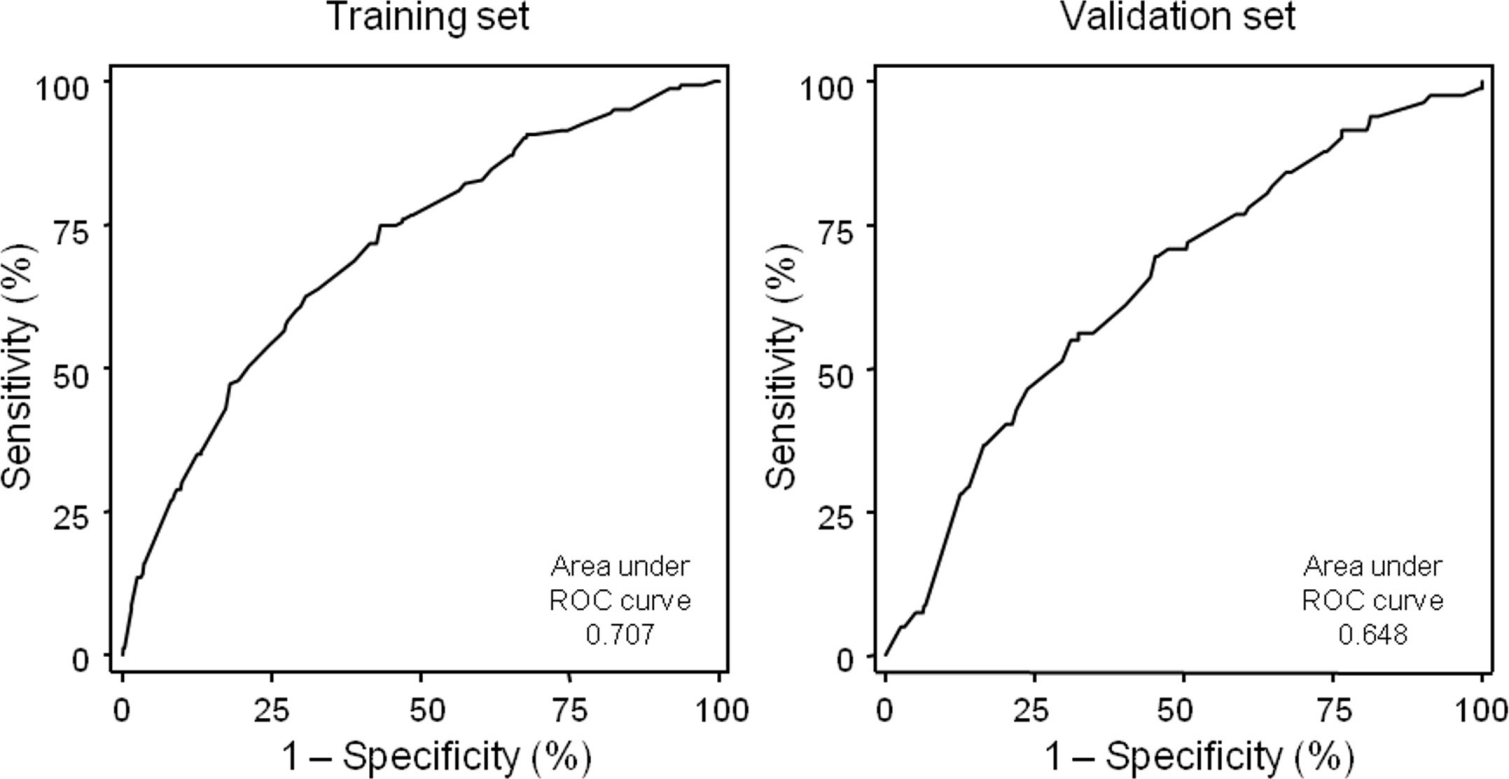
Calibration plots in the training and validation sets.

Elective surgery was associated with a reduced risk of potential DRP (adjusted odds-ratio (aOR) 0.41, p<0.001), as well the main diagnosis of disease of circulatory system (aOR 0.57, p = 0.035). Elective surgery was the reason for hospitalization in 1064 (63.1%) patients. Heart rate ≥ 80 beat per minute (bpm) increased the risk of DRP (aOR 1.41, p = 0.053) and was observed in 724 (42.9%) patients. The risk of potential DRP was increased in patients who had two or more drugs of ATC class A (alimentary tract and metabolism) prescribed on the second day of hospitalization (aOR 3.52, p<0.001), observed in 1055 patients (62.6%), and one or more on day one (aOR 2.24, p = 0.003), which was observed in 583 (34.6%) patients, who were prescribed with a drug of ATC class J (antiinfectives for systemic use) in day one (aOR 1.97, p = 0.001), observed in 386 (22.9%) patients, and in patients prescribed with seven or more drugs in day 2 (aOR 1.63, p = 0.022), with was seen in 1002 (59.4%) patients. The final predictive model was applied to the validation set for determination of the model fit and predictive performance in a distinct set of patients. Model calibration was adequate as assessed by the Hosmer-Lemeshow test (p = 0.57). The calibration plot shown in Fig 2 obtained in the training and in the validation set with 562 patients shows excellent fit of the model to the data in the training set (intercept -0.001, slope 1.002) with some loss of calibration in the training set (intercept -0.033, slope 0.589).

**Fig 3:**
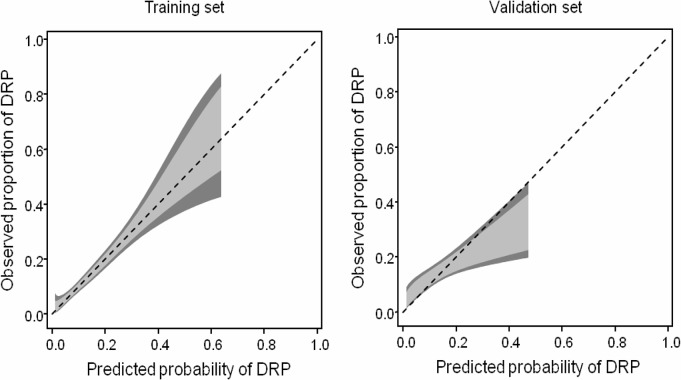
Calibration belt in the training and validation sets. The light grey area represents the 80% confidence bands and the dark grey areas the 95% confidence bands.

Fig 3 shows the calibration belt in the training and the validation sets, again showing good fit of the model to the data in the raining set. In the validation set the calibration belt show that the model somehow overestimates the risk of potential DRP in patients with higher risk.

**Fig 4:**
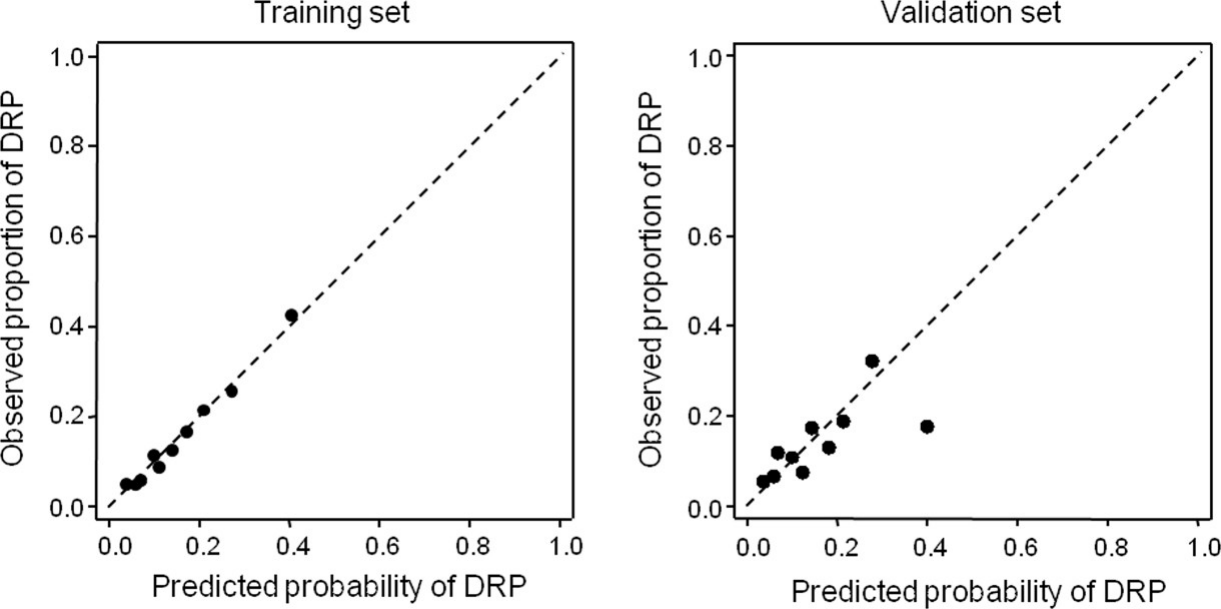
ROC curves of the predictive model obtained in the training and in the validation sets.

Model discrimination, assessed by the area under the ROC curve, was 0.71 (95% confidence interval (CI) 0.66–0.75) in the training set and 0.65 (95% CI 0.58–0.71 in the validation set. Fig 4 presents the ROC curves in the training and in the validation sets.

The Brier score was 0.11 indicating adequate predictive performance of the model. Table 5 presents the sensitivity, specificity and predictive values of the model in the training and the validation sets setting as classification cut-off a predicted probability of potential DRP of 14.6%.

**Table 5:** Model performance in the training and the validation sets.

Statistic	Training set			Validation set		
	n	%	95% CI	n	%	95% CI
Sensitivity	104/163	63.8	55.9–71.2	46/82	56.1	44.7–67.0
Specificity	646/961	67.2	64.1–70.2	313/480	65.2	60.8–69.5
Positive predictive value	104/419	24.8	20.8–29.2	46/213	21.2	16.0–27.4
Negative predictive value	646/705	91.6	89.3–93.6	313/349	89.7	86.0–92.7
Correctly predicted	750/1124	66.7	63.9–69.5	359/562	63.9	59.8-67-9

## Discussion

The present study was based on a large prospective cohort of patients randomly selected among all adult patients admitted to a general tertiary care hospital during a two-year period, excluding patients under chemotherapy and immunosuppression. Several risk factors of potential drug-related problems were identified among patients variables collected at admission and among medicines prescribed in the first two days of hospitalization, with a set of seven independent risk factors identified by multivariate analysis. A multivariate model based on those risk factors demonstrated good fit to the data and fair predictive performance considering that the predicted outcome has low incidence in this patient population.

The most common potential DRP in our study, “effect of drug treatment not optimal”, relate to dosage inadequacy, especially an interval of administration of drug doses shorter than recommended. The occurrence of these errors may be related to insufficient knowledge about newer drugs and clinical protocols by prescribers as a consequence of our hospital being a university hospital, with constant admission of interns and residents with limited experience in pharmacotherapy. Another common DRP in our findings refers to “high treatment costs”, which were due to excessive length of treatment duration, often with antimicrobials in situations of therapeutic ineffectiveness. As noted by, Saokews et al., the use of antimicrobials without proper monitoring results in increased costs, as well as in therapeutic ineffectiveness and patient risk [31].

Although the identification of the patients at risk of events involving drug therapy among adult patients hospitalized in general hospitals is of unquestionable clinical importance, to the best of our knowledge only one observational study has been published with that aim [13]. In that study, Urbina et al. searched for risk factors of DRP using the data of a prospective cohort of 8713 patients admitted to a tertiary care university hospital during a six-month period. Potential DRP were detected both by an alert system coupled with a computerized medical record with an integrated physician order entry and by manual review of all medication orders by a team of clinical pharmacists. They identified several independent risk factors of one or more DRP, namely age, Charlson’s Comorbidity index, polypharmacy, several diagnostic categories (circulatory, digestive, musculoskeletal and connective, kidney and urinary tract) and drugs in several ATC classification classes (cardiovascular system drugs, systemic hormones, systemic antiinfectives, sensory organs agents, and various). The study included an initial validation of the identified predictor variables in 4058 subsequent admissions, reporting an area under the ROC curve of 0.78, with a second validation [27] being published later, based on retrospective cohort of 52,987 patients from the same hospital observed over 4 years, reporting an area under the ROC curve of 0.75. Our study adds to the knowledge base of risk factors of DRP in hospitalized patients by corroborating some of the results of the Urbina study. Several of those independent risk factors correspond or are related to risk factors identified in univariate analysis in our study such as older age, polypharmacy, antiinfectives use. Our study also identified digestive system drugs as important risk factors, with ondansetron and metoclopramide responsible for an important number of DRP. Therefore, the present study also strengthens the notion that risk stratification systems of DRP in patients hospitalized in general hospitals may be successfully developed. One important difference between the two studies, though, is that near one fourth of the potential DRP in the Urbina study were prescription errors related to inadequate utilization of the computerized physician order entry system by the physicians, which may limit the generalizability of the findings, whereas in our study prescription errors related to the use of a computational tool were not that important.

Other studies have presented results on related topics, but none on risk factors of potential DRP in patients in general hospital. Some studies have reported risk factors of adverse drug reactions or adverse events [32–34]. Other studies have described risk factors of potential DRP in specific patient populations, such as a study in a retrospective cohort of 200 patients with rheumatoid arthritis that identified polypharmacy, multiple comorbidities, hyperlipidaemia, renal failure and osteoarthritis as risk factors [35], and a prospective study conducted in 842 patients hospitalized in cardiology wards that concluded that female sex, first hospital admission and each additional medication received increased the risk factors of DRP [25].

In our study, the analysis of variables on hospital admission indicated that elective surgery admission had lower risk of potential DRP, most likely due to a shorter length of stay, less comorbidities, lower complexity of the processes of care and, consequently, fewer medication orders. In contrast, recurrent hospitalizations within the same year, indicating a need to care of previously unresolved and complex health problems, as well as Charlson’s comorbidity index ≥ 5 are related to increased complexity of care [36]. Patient variables such as body mass index below the normal range, increased heart rate, anemia, and leukocytosis are clinical signs common to several serious conditions, especially infections and blood abnormalities [37].

Upon admission and within the next 48 hours, risk factors related to the occurrence of potential DRP include seven or more prescribed medicines, potential drug-drug incompatibilities in the medication order, prescription of drugs administered by the intravenous and subcutaneous routes, and specific ATC classes (alimentary tract and metabolism agents, systemic hormonal preparations, systemic antinfectives, musculo-skeletal, and antiparasitic drugs). Polypharmacy, consisting of the prescription of seven or more medications, predisposes to drug interactions and adverse drug events, especially in patients with comorbidities [38]. Drug-drug incompatibilities are, by definition, cause of DRP. In multivariate analysis drug-drug interactions were no longer statistically significant probably because the drugs more often involved in drug-drug-incompatibilities were alimentary tract agents, which are more markedly associated to the occurrence of DRP. Antiparasitic drugs refer mostly, in the ATC classification, to intravenous metronidazole used for the treatment of infections caused by anaerobic bacteria.

The administration of a single alimentary tract and metabolism agent on admission is associated with decreased risk of potential DRP, possibly because this is routine in patients admitted to elective surgery. However, when controlling for elective surgery in multivariate analysis, the prescription of one medication of that ATC class in day 1, or two or more in day 2, increases the risk of potential DRP.

The seven independent predictors of DRP were selected by multivariate analysis from over 123 candidate variables covering patient demographic, clinical and pharmacotherapeutic data collected within the initial 48 hours upon hospital admission. Many of the study variables overlap the variable set reported in the study by Urbina et al [13] but one important distinction is that we collected, and analysed separately, data on medications prescribed on the first and second hospitalization day. The rationale behind this approach was because the medication prescribed upon patient admission, before the results of several diagnostic exams are known, is often changed the next day when the clinical problems are more accurately identified. The analysis of model calibration and discrimination was in line with the results reported by Urbina et al. [13], the validation of the model in an independent sample of patients indicated that the model is robust but, although sensitivity and specificity values are interesting, they are not in the ideal range for clinical utilization. Therefore, continuing efforts in this line of research are necessary to develop models with better predictive properties.

This study has some limitations. Data were obtained from a single institution, which may somehow prevent the generalization of our findings. In addition, because potential DRPs have been identified from hospital pharmacy records and medical reports, there may be underreporting in view of the method used to identify potential DRPs. The lack of support from an effective computerized warning system of potential DRP and the absence of the clinical pharmacist at patient bedside visits may also have led to under-detection of potential DRP.

However, considering the large sample size and the very limited literature on risk factors of DRP among patients admitted to a general hospital, we believe that the results are quite relevant. The prospective cohort design, the adoption of standard classification system of DRP, and review of 100% of the medication orders by a team of clinical pharmacists are methodological aspects of the study that contribute to the validity of the results.

The identification of predictors of potential DRP is of great clinical value for the selection of patients requiring preventive measures to improve medication safety and quality of care. The development of risk stratification tools to support the work of the healthcare team, especially the clinical pharmacist, will most likely allow the optimization of time and resources. Risk stratification directs drug-related harm prevention strategies, thereby reducing morbidity and mortality, costs, treatment time and, consequently, improving the quality of care.

Future studies should search for additional risk factors of DRP in different hospital settings, including laboratory parameters, focusing not just on variables collected at patient admission but preferably throughout the entire hospital stay.

## Conclusion

The risk of one or more potential DRP during hospitalization in a general hospital was found to be increased in patients with increased heart rate, patients prescribed in the second hospital day with two or more agents acting on the alimentary tract and metabolism or with a systemic anti-infective, and patients administered 2 or more agents acting on blood or blood products during the first two days, while patients admitted for elective surgery have lower risk of potential DRP. The assessment of these risk factors within the first 24 hours of hospitalization may help in the identification of patients at risk for DRP, and may enable clinical pharmacists to guide and target preventive measures in order to limit the occurrence of manifest DRP.

## Supporting information

S1 Data (XLSX)
