# Correction: Risk factors for drug-related problems in a general hospital: A large prospective cohort

**DOI:** 10.1371/journal.pone.0303708

**Published:** 2024-05-08

**Authors:** Valdjane Saldanha, Ivonete Batista de Araújo, Sara Iasmin Vieira Cunha Lima, Rand Randall Martins, Antonio Gouveia Oliveira

The images for Figs 2 and 4 are incorrectly switched. The image that appears as Fig 2 should be Fig 4, and the image that appears as Fig 4 should be Fig 2. The figure captions appear in the correct order.

In the PDF, Fig 3 is incorrectly formatted and partially cropped.

Please see the complete, correct Figs 2, 3 and 4 here.

**Fig 2:**
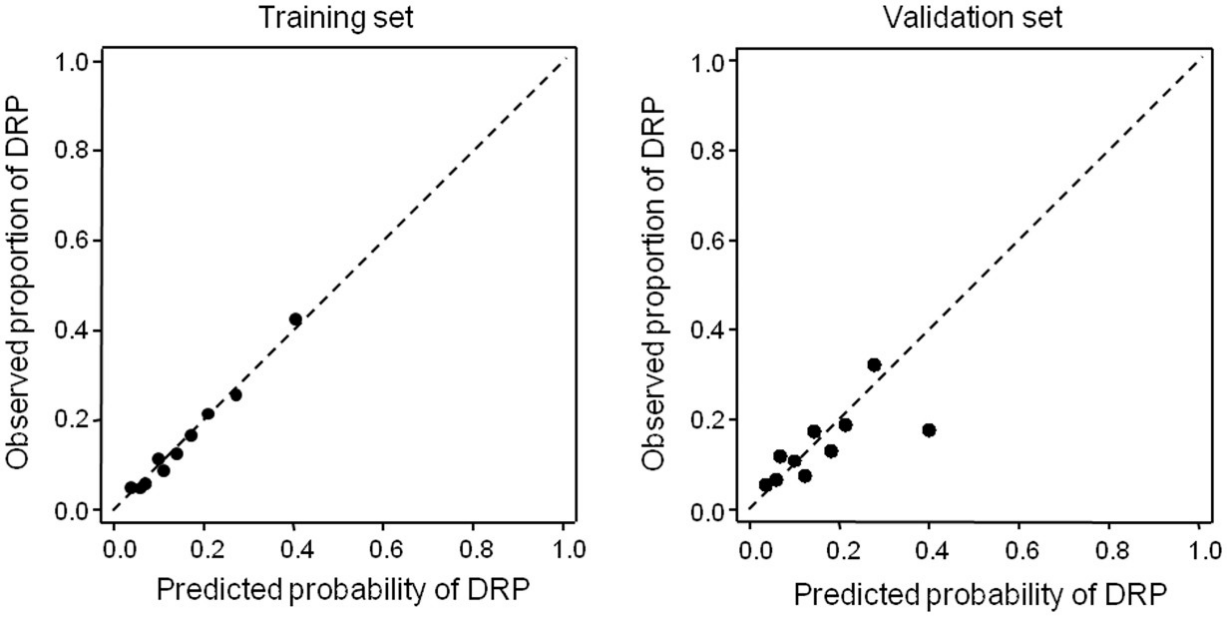
Calibration plots in the training and validation sets.

**Fig 3:**
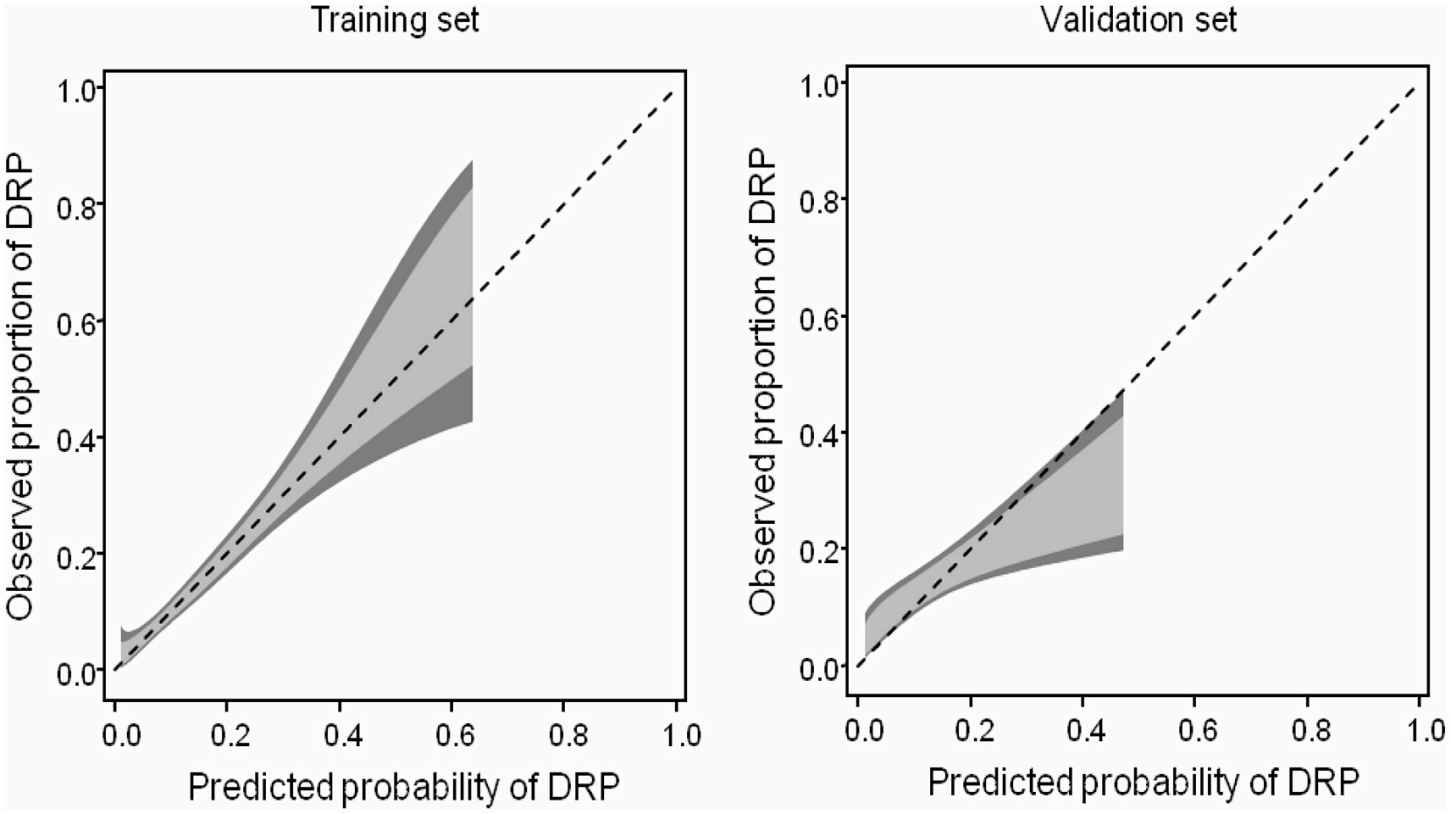
Calibration belt in the training and validation sets. The light grey area represents the 80% confidence bands and the dark grey areas the 95% confidence bands.

**Fig 4:**
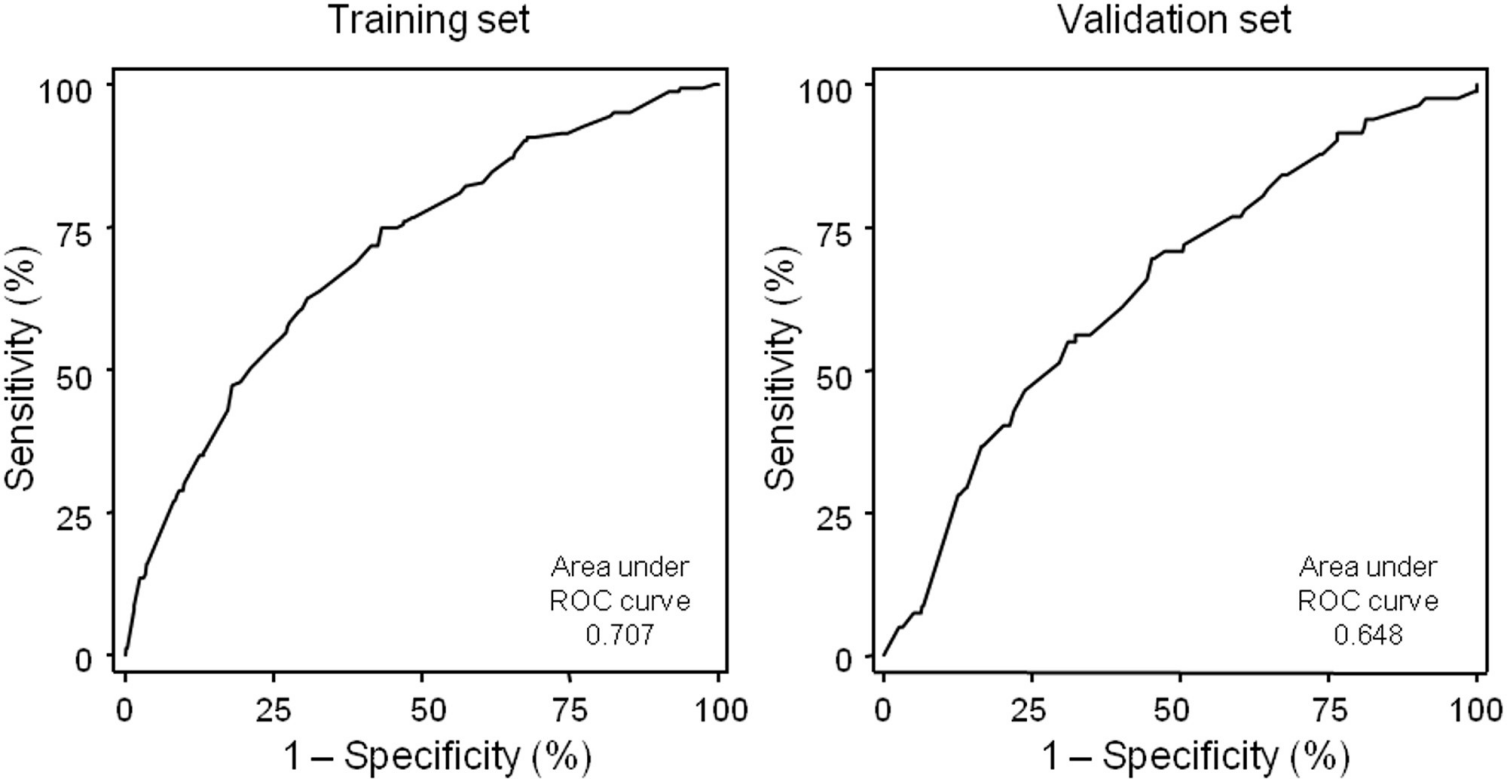
ROC curves of the predictive model obtained in the training and in the validation sets.

In the Patients and methods section, the first sentence of the third paragraph is missing a period. The correct sentence is: In order to obtain the target sample size distributed evenly over the defined two-year patient accrual period, a random sample of patients was obtained by enrolling into the study all eligible patients admitted on two different days (consecutive or not) of each study week.

In the Results section, the word “training” is misspelled in the first sentence of the antepenultimate paragraph. The correct sentence is: Fig 3 shows the calibration belt in the training and the validation sets, again showing good fit of the model to the data in the training set.

There are errors in the author contributions. The correct contributions are:

**Conceptualization**: Valdjane Saldanha, Antonio Gouveia Oliveira.

**Data curation**: Rand Randall Martins.

**Formal analysis**: Antonio Gouveia Oliveira.

**Investigation**: Sara Iasmin Vieira Cunha Lima.

**Methodology**: Valdjane Saldanha, Ivonete Batista de Araújo, Antonio Gouveia Oliveira.

**Project administration**: Valdjane Saldanha.

**Supervision**: Valdjane Saldanha, Ivonete Batista de Araújo, Sara Iasmin Vieira Cunha Lima.

**Validation**: Antonio Gouveia Oliveira.

**Visualization**: Rand Randall Martins.

**Writing – original draft**: Valdjane Saldanha, Ivonete Batista de Araújo, Antonio Gouveia Oliveira.

**Writing – review & editing**: Rand Randall Martins, Antonio Gouveia Oliveira.
